# An anti-Shiga toxin VHH nanobody multimer protects mice against fatal toxicosis when administered intramuscularly as repRNA

**DOI:** 10.1128/iai.00239-24

**Published:** 2024-10-11

**Authors:** Sally R. Robinson, Denise Ann Dayao, Jhon A. Medina, Cara J. Martone, Anne K. Yauch, Troy Hinkley, Jesse H. Erasmus, Charles B. Shoemaker, Saul Tzipori

**Affiliations:** 1 Department of Infectious Disease and Global Health, Cummings School of Veterinary Medicine, Tufts University, North Grafton, Massachusetts, USA; 2 HDT Bio Corp., Seattle, Washington, USA; St. Jude Children's Research Hospital, Memphis, Tennessee, USA

**Keywords:** *E. coli* O157, Shiga toxins, Stx treatment, Stx mouse toxicity model

## Abstract

Hemolytic uremic syndrome (HUS) is a systemic sequelae from gastrointestinal infection with Shiga toxin (Stx) producing *Escherichia coli* (STEC) that can result in acute kidney injury, lasting renal disease, and death. Despite a window for intervention between hemorrhagic diarrhea and onset of HUS, no specific therapies exist to prevent or treat HUS following STEC infection. Furthermore, there is no way to predict which patients with STEC will develop HUS or any rapid way to determine which Stx variant is present. To address this, we have broadened the therpay to neutralize additional toxin variants. It contains a multimer of nanobodies derived from camelid heavy chain antibody fragments (VHHs). An improved VHH-based neutralizing agent (VNA2) is delivered intramuscularly as RNA combined with LION nanoparticles rather than mRNA, that replicates on administration (repRNA), resulting in a rapidly circulating VNA that can bind systemic toxin. The RNA/VNA2-Stx administered intramuscularly prevents toxicity and death in a mouse model of acute Stx toxicity.

## INTRODUCTION

Shiga toxin (Stx)-producing *Escherichia coli* (STEC) infection induces acute diarrhea in humans following ingestion from sources such as contaminated food or water. Infection typically presents as episodes of bloody diarrhea and severe abdominal cramping that is often self-limiting. A serious complication of STEC infection is the development of hemolytic uremic syndrome (HUS), a thrombotic microangiopathy with thrombocytopenia, hemolytic anemia, and acute renal failure which can be fatal or result in long-term renal dysfunction (1).

Shiga toxin (Stx) is the major virulence factor in STEC. It is a bacteriophage-encoded cytotoxin with two major subtypes that are associated with the diarrheal disease: Shiga toxin 1 (Stx1) and Shiga toxin 2 (Stx2) (2). In humans, Stx2 is significantly associated with the incidence of HUS as compared to Stx1- and Stx1/Stx2-producing strains (3, 4). Studies have identified several variants of Stx1 and Stx2, and of these Stx2a is one that has been most frequently associated with HUS (5), and the most extensively investigated.

STEC infection leading to HUS is most common in children under the age of 5 years (6). However, in 2011, a major outbreak of *E. coli-*induced diarrhea with high incidence of HUS among adults (~20% of 4,000 cases) was reported in Europe, most of which occurred in Germany (7, 8). The outbreak was attributed to a potent Stx2 produced by an *E. coli* type not previously associated with STEC; representing an expansion of *E. coli* strains that carry the Stx2 gene (9).

Preventive measures are limited to supportive therapy; antibiotic treatment and antimotility agents are contraindicated as they can lead to serious exacerbation leading to HUS in young children (10, 11). The need for alternative treatments that directly target and neutralize the systemic effects of Stx1 and Stx2 was the focus of our earlier work which demonstrated the protective effect of human monoclonal antibodies (mAbs) in neutralizing Stx and preventing severe disease complications from STEC infections in the gnotobiotic piglet model and fatal intoxication in mice (12–14). However, some challenges of mAb therapies are their short shelf-life, high production cost, and need for intravenous administration (4, 15).

An alternative to the use of mAbs are single-domain antibodies derived from the V_H_ domain of heavy-chain-only camelid antibodies (16), also called VHHs or nanobodies. mRNA delivery of VHHs has potential advantages over human mAb therapeutics. Their small size facilitates customized target-specific VHH molecules that when bound together can potently and broadly target multiple Stx variants. These multimers are termed VHH-based neutralizing agents (VNAs).

When the VNAs are encoded in mRNA, the resulting *de novo* protein expression can produce a robust toxin-neutralizing effect (17, 18). However, the expression of therapeutic levels of biologics from mRNA has so far only been achieved with lipid nanoparticle (LNP) delivery to multiple tissues throughout the recipient due to the broad biodistribution of LNPs (19, 20). As this broad biodistribution is associated with reactogenicity (21), we have instead focused on localized delivery to the injected muscle tissue (22), requiring strategies for enhanced biologics expression. To achieve higher expression from fewer transfected cells *in vivo*, we applied self-amplifying replicon RNA (repRNA) derived from an alphavirus genome (23). These molecules amplify an mRNA encoding the therapeutic of interest, producing 10- to 100-fold more protein compared to conventional mRNA when delivered with a localizing cationic nanocarrer such as LION, developed by HDT Bio (24, 25). As with all cationic nanocarriers, RNA is complexed to the nanoparticle surface, enabling bedside mix just before administration for immunotherapy. Furthermore, the formulated repRNA can be administered intramuscularly and produce significant serum levels of encoded secreted product (18, 25). Using botulinum neurotoxins, we demonstrated that long chains of linked VHHs containing up to six different VHH components could be encoded as repRNA and delivered to mice, eliciting the expression of the VHH heterohexamer in which all component monomers remained functional and the agent was capable of potently neutralizing three different botulinum neurotoxin serotypes (26).

As an alternative to mAbs for treating Shiga toxin producing *E. coli* (STEC) infections, we reported the development of camelid single-domain antibodies (called VHHs or nanobodies) that bind and neutralize Stx1 and/or Stx2 (26). By linking three such VHHs together into a single VHH-based neutralizing agent (VNA), we demonstrated protection in mice against lethal doses of both Stx1 and Stx2 injections. Furthermore, we demonstrated that this heterotrimeric VNA, administered parenterally as a protein or expressed by recombinant adenovirus treatment, protected piglets from the pathology associated with STEC infection in gnotobiotic piglets, a well-established model of human STEC (13). The original VNA consisted of VHH agents that had not been assessed for their specificity to Stx2 natural variants, and we have since found that the Stx2a-binding VHH components were poorly able to bind the important Stx2d variant (27). Upon re-screening of our VHH panels, we identified several Stx2-neutralizing VHHs displaying good affinity for Stx2d. We, thus, created a VHH heterotetramer designed for broader Stx natural variant specificity. Here, we report the testing of the new VNA, encoded in formulated RNA, in a mouse model of Stx2 intoxication.

## RESULTS

### Design of VNA effective on a broad range of Shiga toxin natural variants

**Fig 1:**
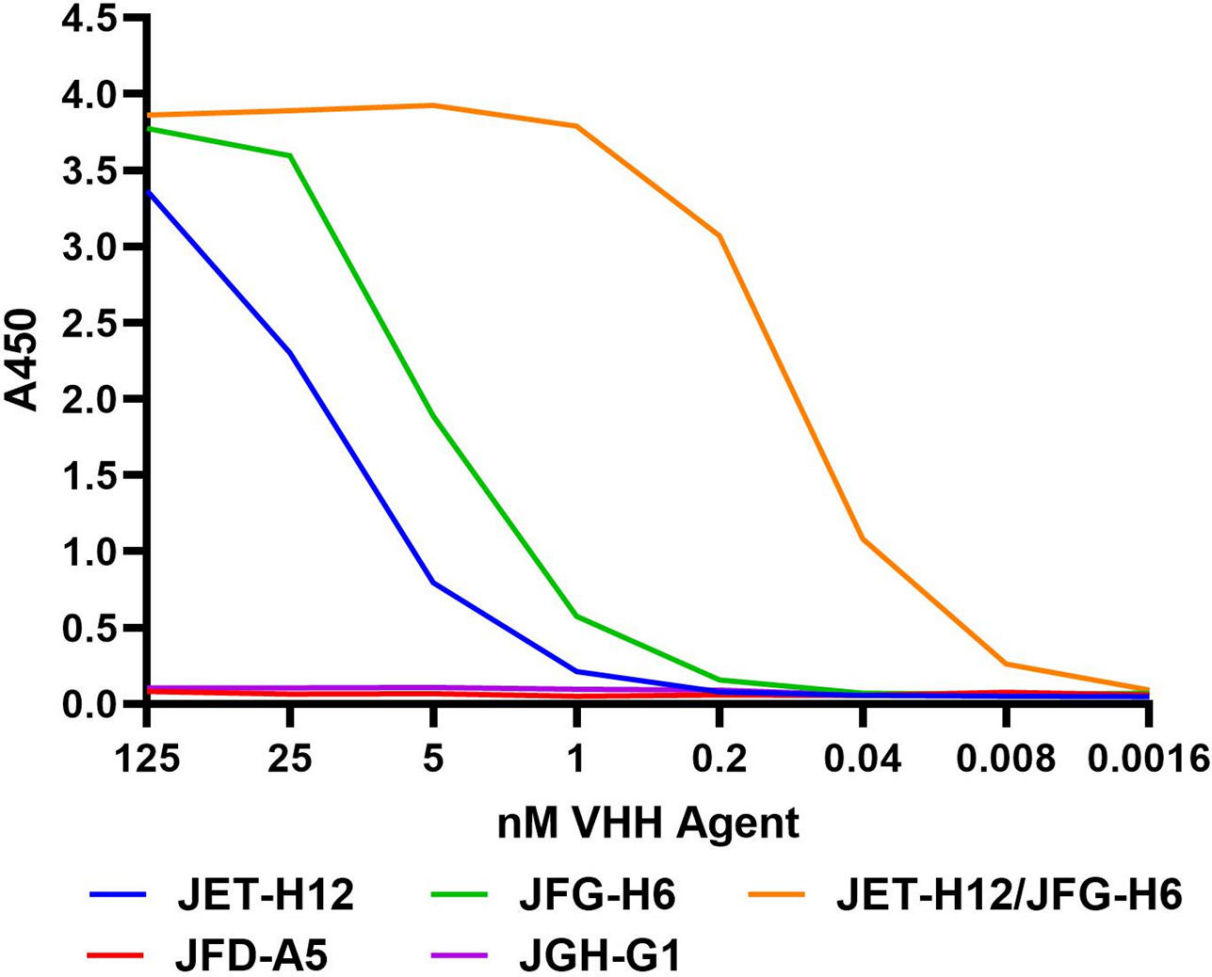
Stx2d-binding ELISA comparing various Stx2a-neutralizing VHHs. Stx2d-binding was assessed using a mAb-capture ELISA as described in Materials and Methods. VHHs or VHH dimers were tested in a dilution series to assess their apparent affinities for Stx2d.

Our previously reported Shiga toxin-neutralizing VNA, A9/A5/G1 (26), here called VNA1-Stx, was demonstrated to potently neutralize both Stx1 and Stx2a (O157) natural variants. More recent analysis has shown that two Stx2a-neutralizing component VHHs of VNA1-Stx, JFD-A5 and JGH-G1, were poor binders of the medically important, and distantly related, Stx2d natural variant (28). We, thus, re-screened our panel of Stx-binding VHHs and identified two VHHs showing good cross-specificity with Stx2a and Stx2d. Figure 1 shows that both JET-H12 and JGH-H6 bind well to Stx2d, and this binding is much improved when the two VHHs are linked into a single protein. JFD-A5 and JGH-G1 display no binding to Stx2d. JET-H12 was, thus, selected to replace JFD-A5, and JGH-H6 was added as a fourth VHH component to create VNA2-Stx. The resulting VHH heterotetrameric, VNA2-Stx, consisted of VHH components JET-A9/JET-H12/JGH-H6/JGH-G1 in this order, each separated by a spacer of five glycine residues.

### STX2A toxicity from systemic administration in mice

**Fig 2:**
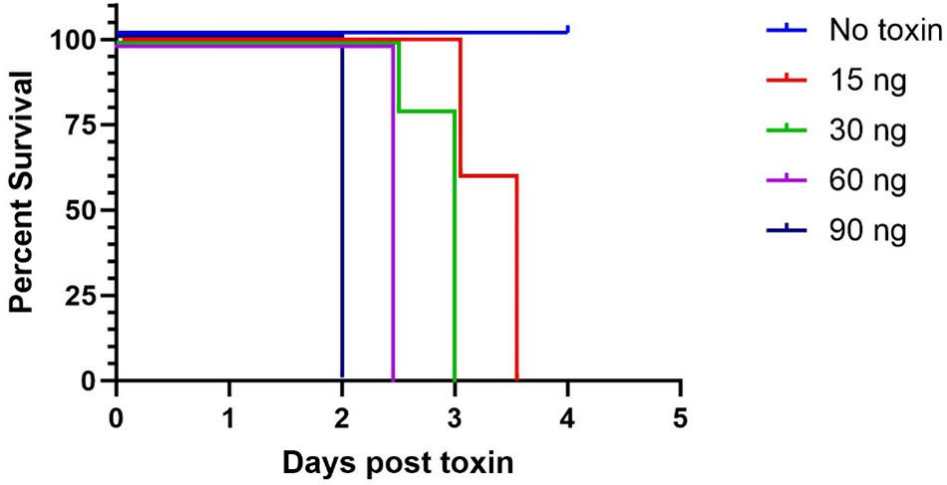
Stx2a toxicity as demonstrated by the probability of survival for mice given a dose ranging from 15 to 90 ng (*n* = 5 mice each). Stx2a was delivered intraperitoneally.

Administration of Stx2a intraperitoneally at doses ranging from 15 to 90 ng results in 100% mortality (Fig. 2). All five mice in the group that received the 15 ng lowest dose reached 15% or greater loss in body weight by the end of day 3. Comparison of survival curves for the other doses indicated a significant difference in survival with time among toxin groups (*P* < 0.0001). This reflects the more uniform and acute mortality with increasing Stx2a dose. We opted to use the 15 ng minimum lethal dose for studies as it provides a longer window to evaluate the efficacy of the therapeutic.

### Mice are protected from STX2A toxicity by RNA-encoded (RNA/VNA2-STX) administration

**Fig 3:**
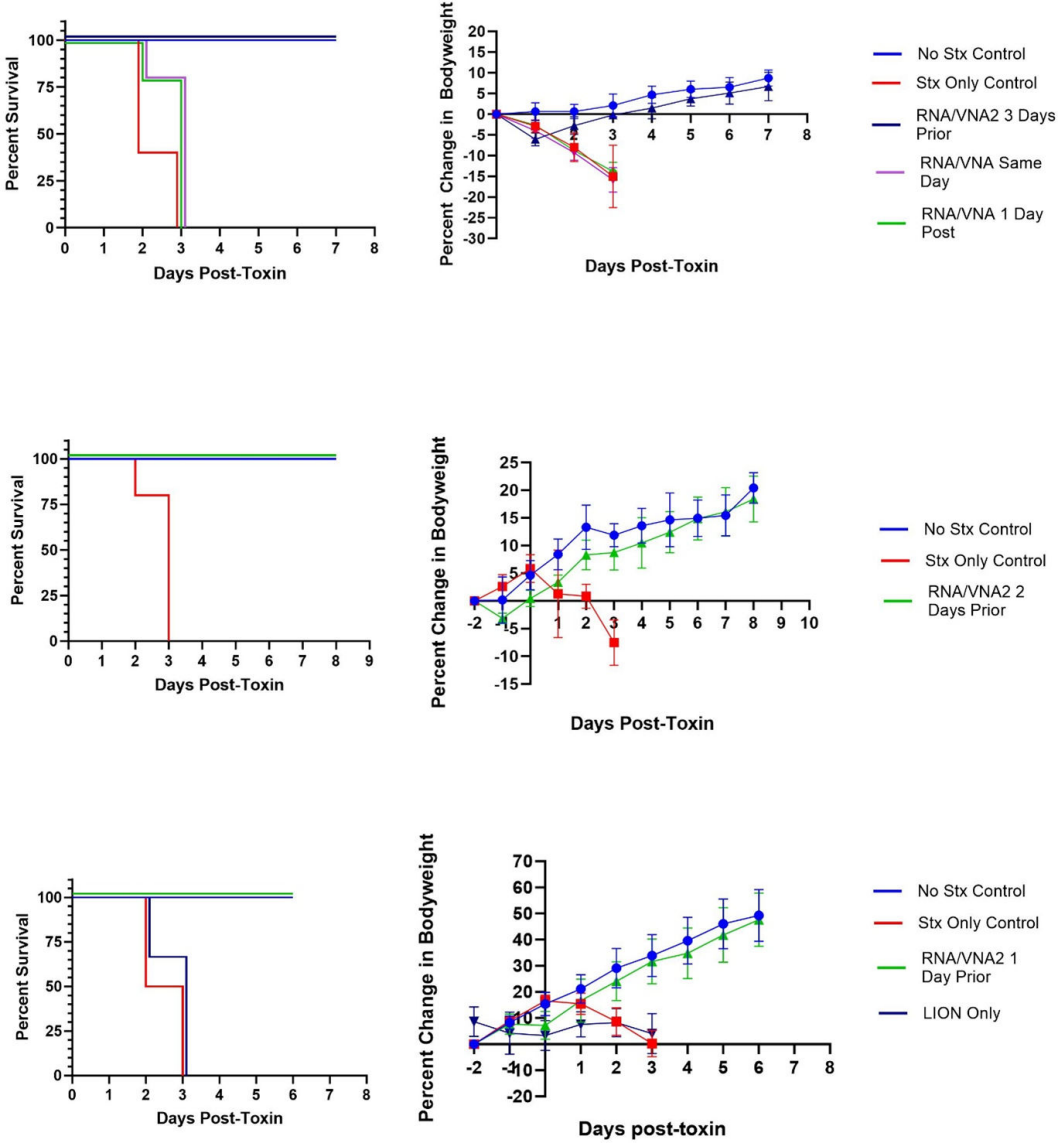
RNA**/**VNA2-Stx delivered intramuscularly at different timepoints relative to Stx2a (15 ng) administration. Comparison of survival (left) and body weight (right) for the three studies: (top) iteration 1, (middle) iteration 2, and (bottom) iteration 3.

**Fig 4:**
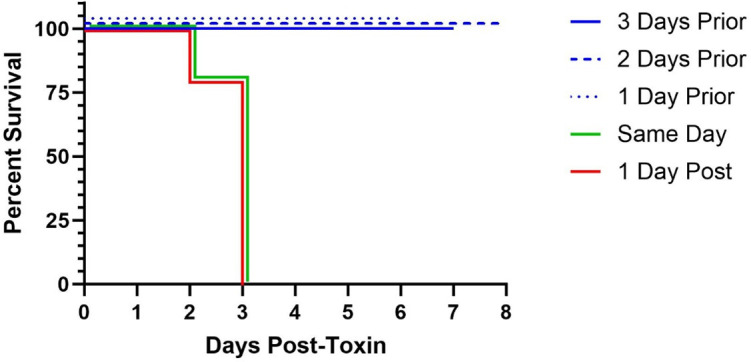
Comparison of survival for RNA/VNA2-Stx administration across the study iterations. Protection from lethal toxicity was dependent on timing of RNA/VNA2-Stx administration in relation to Stx2a challenge. Mice given RNA/VNA2-Stx 3, 2, or 1 day before Stx2a had protection while those given on the same day or 1 day after Stx2a did not survive beyond 3 days post-challenge.

VNA2-Stx was encoded as repRNA (25), formulated with the cationic nanocarrier, LION (25), and administered intramuscularly to mice employing the Stx2a toxicity mouse model in three experimental subsets. The three iterations served to evaluate protective efficacy and window of therapeutic administration in this model of acute toxicity. Survival across all groups the study iterations is shown in Fig. 3, and a breakdown of percent change in body weight by treatment group is summarized in Table 1. Figure 4 shows a comparison of survival based on timing of RNA/VNA2-Stx administration across all studies.

**TABLE 1:** Stx2a study overview

Iteration (duration)	Treatment group (*n*)	RNA/VNA2-Stx administration	Surviving individuals (*n*)	Change in BW at endpoint	SD
1 (7 days)	Negative control (3)	–	3	8.7%	1.9
	Positive control (5)	–	0	−11.8%	5.3
	mRNA treated (2)	3 days pre-Stx2a	2	6.7%	3.4
	mRNA treated (5)	Same day as Stx2a	0	−15.0%	3.1
	mRNA treated (5)	1 day post-Stx2a	0	−12.9%	2.8
	Negative control (5)	–	5	20.4%	2.8
2 (8 days)	Positive control (5)	–	0	−6.2%	4.6
	mRNA treated (5)	2 days pre-Stx2a	5	18.4%	4.1
	Negative control (10)	–	10	49.3%	9.9
3 (6 days)	Positive control (10)	–	0	2.9%	5.8
	Vehicle + toxin (6)	–	0	1.8%	8.0
	mRNA no toxin (10)	–	10	30.1%	5.2
	mRNA treated (10)	1 day pre-Stx2a	10	47.6%	10.2

The first iteration of the mouse experiments compared the positive (intraperitoneal Stx2a) and negative (intraperitoneal PBS) control groups with female mice that were administered intramuscular rRNA/VNA2-Stx either 3 days prior, the same day as, or 1 day after Stx2a challenge. As shown in Fig. 3A, mice given RNA/VNA2-Stx 1 day before or the same day as Stx2a challenge did not survive beyond 3 days post-toxin. This mortality was equivalent to the positive control mice given toxin alone. In contrast, mice treated 3 days prior to Stx2a challenge all survived, as did negative control mice. There was a significant difference in the probability of survival between all groups (*P* = 0.0045). There was also a significant difference in survival when comparing the effect of treatment between the positive control Stx2a mice with RNA/VNA2-Stx group treated 3 days before toxin (*P* = 0.0310). The average percent change in body weight from baseline to the end of the study period or death is shown in Table 1. Mice in the positive control group that received toxin but no treatment lost weight and were humanely euthanized (Fig. 3A). There was no significant difference in body weight between the surviving groups of mice administered RNA/VNA2-Stx 3 days before toxin and the negative control group (*P* = 0.4418).

The second iteration of the mouse experiments with female mice compared the positive (Stx2a) and negative (PBS) control groups with mice given RNA/VNA2-Stx 2 days prior to Stx2a challenge (Fig. 3B). All mice in the positive control group reached humane endpoint of weight loss and were euthanized within 3 days post-toxin. All mice treated with RNA/VNA2-Stx 2 days prior to Stx2a challenge survived the study period. Survival analysis showed there was a significant difference in the probability of survival between groups (*P* < 0.0001), and significant effect of the treatment on survival with direct comparison between the RNA/VNA2-Stx treated toxin vs toxin positive control groups (*P* = 0.0035). The RNA/VNA2-Stx treated mice gained body weight similar to the negative control mice (*P* = 0.4410).

**Fig 5:**
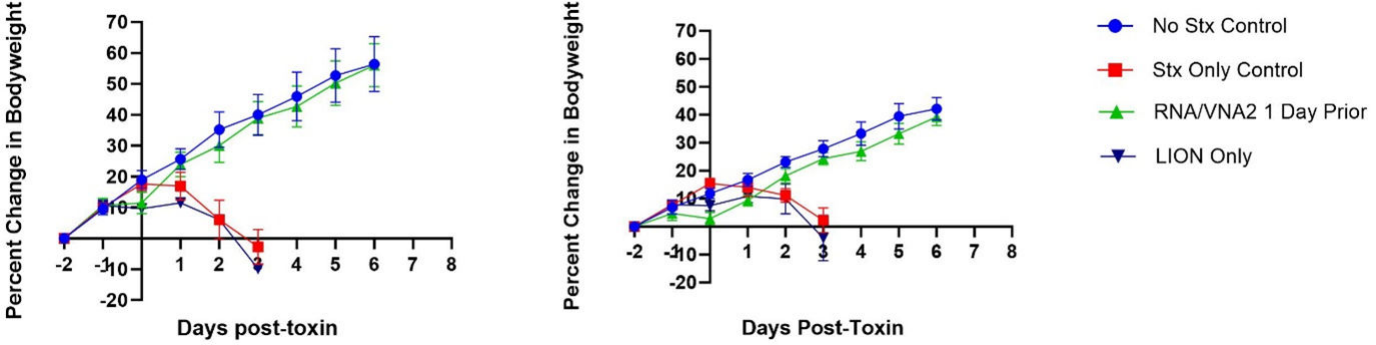
RNA/VNA2-Stx treatment was protective in both male (left) and female (right) mice. Data are from iteration 3 where RNA/VNA2-Stx was administered 1 day before Stx2a challenge.

The third iteration of the mouse experiments (Fig. 3C) compared male and female mice in the positive and negative control groups with mice given either LION vehicle without RNA or RNA/VNA-Stx 1 day prior to Stx2a challenge. A group of mice were administered RNA/VNA2-Stx alone (data in Table 1, but not shown on graphs). Male and female mice in the positive Stx2a control or LION vehicle + Stx2a groups did not survive beyond day 3 post Stx2a challenge. All mice in the negative control and RNA/VNA2-Stx only groups survived the study period. Survival analysis showed there was a significant difference in the probability of survival between groups (*P* < 0.0001). Comparison of the toxin and RNA/VNA2-Stx treated toxin groups also showed a significant difference in survival (*P* < 0.0001). The average percent change in body weight from baseline to end of study period or humane endpoints is shown in Table 1. Body weight (Fig. 3C) was not significantly different between the two surviving groups (negative control and RNA/VNA2-Stx treated mice) (*P* = 0.7147). Body weight curves were similar between male and female mice across treatment groups (Fig. 5).

**Fig 6:**
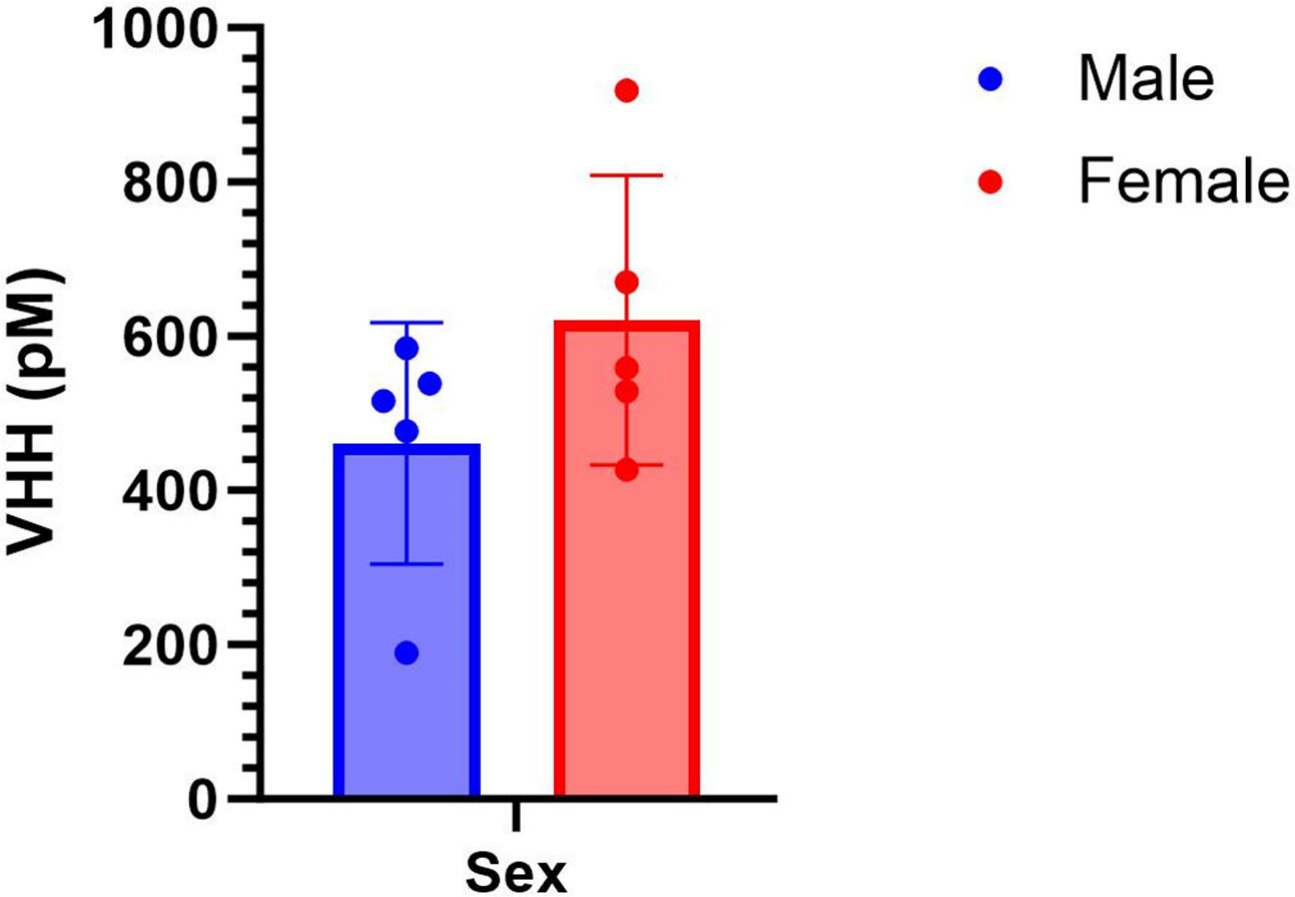
VHH levels in serum quantified by ELISA 4 days after RNA/VNA2-Stx administration. Range 190–919 pM. [VHH] did not differ by sex (*P* = 0.1827).

Mice treated with RNA/VNA2-Stx only had VHH levels in blood sera that ranged from 190 to 919 PM (Fig. 6; mean = 541 pM; SD = 183 pM) 4 days after administration (at the time of peak Stx2a-induced mortality). This low, but detectable, level is consistent with prior studies employing a different repRNA-encoded VNA in which serum VNA expression peaked at day 2 ~10 nM and was undetectable at day 5 (12). VHH levels did not differ by sex (*P* = 0.1827).

The percent change in body weight from baseline for each study by treatment group is shown in Fig. 3. A transient dip in body weight was consistently observed 1 day after RNA/VNA2-Stx administration. Across all studies, mice lost an average of 3.2% of their body weight 1 day after administration of RNA/VNA2-Stx or LION vehicle alone (SD = 2.8); body weight rebounded to baseline level or greater an average of 2.2 days after treatment (SD = 1.1). Overall, there was no significant difference in the probability of survival for male and female mice (*P* = 0.9653).

## DISCUSSION

In the absence of an effective treatment (antibiotics are contraindicated) or control strategies to prevent the development of HUS in children with evidence of infection or exposure to STEC, toxin-neutralizing nanobody multimers known as VHH-based neutralizing agents (VNAs) delivered as a repRNA therapeutic offer a potential alternative. The prodromal interval of 3–7 days between the onset of STEC-mediated diarrhea and development of HUS offers a short window of opportunity for intervention. This led to the development of effective neutralizing recombinant human mAb against Stx1 and Stx2 to be administered IV at onset of diarrhea. Despite monoclonal antibody (mAb) treatments being shown to be highly effective in mice and in piglets, it proved to be impractical due to high cost of production, relatively short shelf-life, and requirement for intravenous administration. In addition, individual mAbs have limited breadth across Stx variants. As STEC cases are in limited numbers, occur sporadically, and there is currently no rapid method for determining the Stx variant present, these limitations make mAbs therapies less feasible (4).

As an alternative to antitoxin mAb, we generated an Stx-neutralizing heterotrimer consisting of three VHHs (26). This VHH-based neutralizing agent (VNA) was highly protective in mice against systemic toxicosis induced by both Stx1 and Stx2a, especially when co-administered with an effector antibody to promote toxin clearance. In a second study, we reported that adenovirus expressing the same VHH heterotrimer VNA prevented piglets from developing systemic fatal disease when given intravenously 48 h after oral challenge with Shiga toxin producing *E. coli* O17:H7 [2013].

For the current study, we developed an improved VNA, called VNA2-Stx, designed to have broader natural variant specificity and at least equivalent potency to the previously reported heterotrimer (26). This modification was motivated by more recent studies demonstrating that the VHH components of the original heterotrimer displayed poor binding to the distantly related, and medically important, Stx2d variant. We, thus, re-screened our VHH display phage library for VHHs showing broad specificity for both Stx2a and Stx2d, and capable of neutralizing both toxins in cell-based assays. We incorporated two of the new VHHs with two previously reported VHHs as components of a new VHH heterotetramer VNA2-Stx to develop an antitoxin that would broadly bind and neutralize Stx1, Stx2a, and Stx2d natural variants.

In addition, for this study, we chose to employ repRNA encoded VNA2-Stx as our means of administration. Our preference was an RNA therapeutic that permits intramuscular (im) administration with a localizing nanocarrier as this modality is safer to patients with reduced systemic toxicity (22) and can be readily performed by medical technicians working in field clinics and doctor’s offices. Since intramascular treatments with conventional mRNA using musle-localized formulations result in relatively low serum levels of encoded secretory proteins (24), we chose to employ a self-amplifying replicon RNA approach known as repRNA that has demonstrated the promise of yielding higher, more sustained expression levels. The cationic nanocarrier formulation employed here for repRNA is LION and was successfully employed previously to (i) express a heterohexameric VNA that protected mice from three different botulinum neurotoxin serotypes (18) and (ii) antiviral monoclonal antibodies (24, 29) that protected mice from viral challenge. Another advantage of LION-formulated repRNA is that the formulation step can be performed at the time of treatment which offers much greater versatility in treatment options as a varied inventory of repRNA products can be inventoried and combined with the LION stock as needed by patients. Finally, this approach permits more rapid introduction of new repRNA products in response to emerging threat agents as compared to products requiring factory formulation.

Beginning with optimizing the Stx2a challenge, 15 ng was shown to consistently induce fatal systemic toxicosis and was used in all subsequent experiments for the evaluation of RNA/VNA2-Stx. Mice given RNA/VNA2-Stx formulated with LION 3, 2, or 1 day before Stx2a administration were fully protected, while those treated on the same day or 1 day after Stx2a did not survive beyond 3 days post-challenge. The delayed protection provided by RNA/VNA2-Stx contrasts with the immediate onset of protection offered by recombinant human mAb which is immediately available upon intravenous administration. Some delay in protection with RNA/VNA2-Stx is attributable to the time required for replication and protein synthesis in the host cells to generate protective levels of circulating VNA. While we quantified levels of VHH in serum 4 days after administration—at the time when treated mice were protected against mortality—the minimum concentration required for protection has not been evaluated. The low serum VNA levels are consistent with prior studies in which we treated mice with a repRNA-encoded VNA that neutralizes Botulinum neurotoxins (BoNT) and found serum levels peaked at ~10 nM on day 2 and became undetectable by day 5 (12). Despite these low levels, mice were protected from exposure to 100 LD_50_ of BoNT. Protection at such low levels is not surprising as lethal levels of Stx2 or BoNT are sub-nM, and other studies have found that VNAs are effective at near equimolar levels (30). The uptake of systemically administered Stx into cells is rapid, and the Stx exerts its toxicity by interfering with protein synthesis. Stx-neutralizing therapies, thus, need to bind toxin and inhibit binding and entry into cells to prevent toxicosis.

Mice treated with the RNA/VNA2-Stx formulation consistently exhibited loss of weight 1 day after administration with rebound and return of weight after an average of 2.2 days. The RNA/VNA2-Stx formulation may induce an innate immune response leading to a reduction in food and water intake, but we have not evaluated a mechanism for the transient loss in body weight.

## MATERIALS AND METHODS

### ELISAS

Dilution ELISAs were performed basically as previously described (26). For Stx2d ELISAs, plates were first coated with 2 µg/mL of the previously reported anti-Stx2 mAb, 5C12 (12) and then, after blocking, the plates were incubated with 1 µg/mL of Stx2d (Phoenix Labs, Tufts Medical Center, Boston, MA). Binding of various, previously described, anti-Stx2a-binding VHHs as monomers or dimers (26), each expressed with an E-tag, was tested by performing a 1:5 dilution series starting at 125 nM. Bound VHH was detected with HRP-coupled goat anti-E-tag (Bethyl).

### Development of VHH

The VHH tetramer employed in this study consisted of four different VHH components, each binding to either or both Stx1 and Stx2. A five amino acid spacer (GGGGG) separated each of the VHH components. The VHH components, from amino to carboxyl end, were JET-A9, JFG-H6, JET-H12, and JGH-G1. The characterizations for three of these VHHs were previously reported (26). JET-H12 possesses properties very similar to the previously reported VHH, JFD-A5, as both show 1–10 nM affinity for the B subunits on both Stx1 and Stx2a, and both weakly neutralize these toxins as monomers, but JET-H12 also displayed low nM binding affinity for Stx2d. JET-H12 also displayed the same strong neutralizing potencies when fused to a second Stx2-neutralizing VHH as was previously reported for JFD-A5 (26).

### Preparation of RNA/VNA2-STX for administration to mice

The RNA/VNA2-Stx formulation was prepared from repRNA stored at −80 and diluted in RNAse-free water. LION stored at 4°C was prepared in a mixture of 40% sucrose, 100 mM citrate, and RNAse-free water. The RNA and LION solutions were combined and incubated at room temperature for a minimum of 15 min before being stored on ice. The formulation was drawn into insulin syringes and administered intramuscularly with animals under isoflurane anesthesia in four 50 µL doses per mouse, giving a total dose of 40 µg RNA per mouse.

### Preparation of Stx for *in vivo* injections

Shiga toxin subtype 2a (Stx2a) was produced in *E. coli* by the Special Studies Lab at Tufts Medical Center (22). Lyophilized Stx2a was prepared to 1 mg/mL stock in water and stored at −80°C. Stock was thawed on ice and diluted in PBS to the desired concentration to be administered to mice in a volume of 50 µ intraperitoneally.

### *In vivo* mouse study of Stx2a dose-response

To establish the optimal challenge dose, 4-week-old female Swiss Webster mice (Charles River Laboratories) were weighed and distributed into groups of five mice each ensuring each group had approximately the same group weight. Mice were administered Stx2a via 100 µL intraperitoneal (IP) injection at a dose of 15, 30, 60, or 90 ng ; a control group of mice was administered equal volume of PBS IP. To determine the minimum lethal dose (MLD), body weights (BW) were measured daily, and mice were monitored for clinical signs of toxicity, including trembling, ataxia, paralysis, and opisthotonos. Mice exhibiting CNS signs or a 15% or greater loss in body weight from their highest weight were humanly euthanized. All surviving mice were euthanized 7 days post-toxin administration.

### Evaluation of VHH protection against Stx challenge in mice

Male and female 3- to 4-week-old Swiss Webster mice (Charles River Laboratories) were weighed and distributed into groups of three to five individuals based on age, sex, and approximate same group weight. Mice in the toxin groups were administered a dose of 15 ng Stx2a in 100 µL via IP injection. For the treatment groups, mice were given 40 µg of the RNA/VNA2-Stx formulation via four 50 µL intramuscular (IM) injections. A positive control group consisted of mice given Stx2a without any treatment, and negative control groups consisted of mice given PBS with or without RNA/VNA2-Stx treatment. A vehicle-only group of Stx2a mice was treated with LION alone. Treatment groups were administered RNA/VNA2-Stx at different timepoints in relation to Stx2a challenge (3 days before, 2 days before, 1 day before, same day, or 1 day after). Body weights were measured daily, and mice were monitored for clinical signs of toxicity for 6–8 days following Stx2a challenge. Mice exhibiting CNS signs or 15% or greater loss in body weight from their highest weight were humanly euthanized. A subset of mice given RNA/VNA2-Stx without Stx2a challenge were sacrificed 4 days after treatment administration, and blood serum was collected for measurement of VHH levels.

### Quantification of anti-STX VHH in biological samples

Enzyme-linked immunosorbent assays (ELISAs) were performed to assess levels of anti-Stx VHH heterotetramer in blood sera against an anti-Stx VHH heterotetramer (JKB-2). After collection, blood samples were centrifuged at 5,000 rpm for 8 min; serum was collected and stored at −20°C. High binding ELISA plates (Greiner, Microlon 655061) were coated with 1 µg/mL of Stx2a in PBS followed by a blocking buffer consisting of 4% nonfat dry milk powder with 0.1% Tween-20 in PBS. Samples were thawed at room temperature and then serially diluted in blocking buffer and incubated overnight at 4°C. Bound agent was detected with 1:2,000 rabbit-α-VHH polyclonal serum (produced in house (31), incubated at room temperature for 1 h, washed, and detected with goat-α-rabbit IgG-HRP (Southern Biotech, 4010-05) incubated for 1 h at room temperature. HRP was quantified by tetramethylbenzidine (TMB), and absorbance was measured at 450 nm with a Biotek Synergy HTX multi-mode reader. Standard curves plotted as non-linear polynomial with blank A450 against JKB-2 concentration in Gen5 software to calculate quantity of VHH in samples. All standards and samples were performed in duplicate.

### Statistical analyses

All statistical analyses were performed using GraphPad Prism version 10 (Graphpad) and Stata/SE 16.1 (StataCorp). Survival curves were assessed using the Mantel-Cox log-rank test. For comparisons of body weight change, the Kruskal-Wallis test by ranks was used when comparing between multiple treatment groups and the independent samples *t*-test was used when comparing across sexes. For all analyses, a *P*-value less than or equal to 0.05 was the cutoff for determining statistical significance.
